# Efficacy and safety of an innovative short-course regimen containing clofazimine for treatment of drug-susceptible tuberculosis: a clinical trial

**DOI:** 10.1080/22221751.2023.2187247

**Published:** 2023-03-17

**Authors:** Xubin Zheng, Xuwei Gui, Lan Yao, Jun Ma, Yifan He, Hai Lou, Jin Gu, Ruoyan Ying, Liping Chen, Qin Sun, Yidian Liu, Chih-Ming Ho, Bai-Yu Lee, Daniel L. Clemens, Marcus A. Horwitz, Xianting Ding, Xiaohui Hao, Hua Yang, Wei Sha

**Affiliations:** a Clinic and Research Centre of Tuberculosis, Shanghai Key Laboratory of Tuberculosis, Shanghai Pulmonary Hospital, Tongji University, Shanghai, People’s Republic of China; b Department of Mechanical and Aerospace Engineering, University of California, Los Angeles, CA, USA; c Department of Bioengineering, University of California, Los Angeles, CA, USA; d Division of Infectious Diseases, Department of Medicine, University of California, Los Angeles, CA, USA; e Institute for Personalized Medicine, School of Biomedical Engineering, Shanghai Jiao Tong University, Shanghai, People’s Republic of China

**Keywords:** Short-course treatment, clofazimine, parabolic response surface, pulmonary tuberculosis, randomized clinical trial

## Abstract

In preclinical studies, a new antituberculosis drug regimen markedly reduced the time required to achieve relapse-free cure. This study aimed to preliminarily evaluate the efficacy and safety of this four-month regimen, consisting of clofazimine, prothionamide, pyrazinamide and ethambutol, with a standard six-month regimen in patients with drug-susceptible tuberculosis. An open-label pilot randomized clinical trial was conducted among the patients with newly diagnosed bacteriologically-confirmed pulmonary tuberculosis. The primary efficacy end-point was sputum culture negative conversion. Totally, 93 patients were included in the modified intention-to-treat population. The rates of sputum culture conversion were 65.2% (30/46) and 87.2% (41/47) for short-course and standard regimen group, respectively. There was no difference on two-month culture conversion rates, time to culture conversion, nor early bactericidal activity (*P *> 0.05). However, patients on short-course regimen were observed with lower rates of radiological improvement or recovery and sustained treatment success, which was mainly attributed to higher percent of patients permanently changed assigned regimen (32.1% vs. 12.3%, *P *= 0.012). The main cause for it was drug-induced hepatitis (16/17). Although lowering the dose of prothionamide was approved, the alternative option of changing assigned regimen was chosen in this study. While in per-protocol population, sputum culture conversion rates were 87.0% (20/23) and 94.4% (34/36) for the respective groups. Overall, the short-course regimen appeared to have inferior efficacy and higher incidence of hepatitis but desired efficacy in per-protocol population. It provides the first proof-of-concept in humans of the capacity of the short-course approach to identify drug regimens that can shorten the treatment time for tuberculosis.

## Introduction

Tuberculosis (TB) continues to be a major public health problem with an estimated new cases of 10 million and nearly 1.5 million death globally in 2020 [1]. As a curable infectious disease, improving its clinical prognosis has long been a hot topic of research. Shortening the course of TB treatment, while ensuring non-inferior efficacy and non-relapse, can bring multiple benefits, including reduced risk of transmission and improved adherence to treatment. In recent years, increasing efforts have been made to shorten the treatment duration for drug-susceptible TB [2,3]. A new four-month regimen (2HPMZ/2HPM) is now recommended in the latest World Health Organization (WHO) treatment guidelines [4,5]. However, its widespread use might be hindered in the regions with concerns on access to rifapentine [6].

As a repurposed drug, clofazimine has been reported with delayed but potent bactericidal activity against *Mycobacterium tuberculosis* (*M. tuberculosis*) and has been involved in many *in vivo* studies on shortening the duration of anti-TB treatment [7–10]. To enrich the arsenal of treatment regimens, we have designed an original all-oral four-month regimen for the treatment of patients with primary drug-susceptible TB based on innovative concept using an output-driven approach by Parabolic Response Surface (PRS). This new regimen, consisting of clofazimine, prothionamide, pyrazinamide and ethambutol, was shown to outperform the current standard regimen in an *in vitro* macrophage model and reduce the time needed to achieve relapse-free cure in *M. tuberculosis*-infected mice [11,12]. Therefore, we undertook a pilot, open label, randomized clinical trial to preliminarily assess the efficacy, antimicrobial activity, tolerability and safety of this new PRS regimen in patients with newly diagnosed bacteriologically-confirmed pulmonary TB in Shanghai, China.

## Materials and methods

### Trial design and participants

This was a prospective, randomized, parallel group, controlled, open-label pilot clinical trial conducted at the Shanghai Pulmonary Hospital, one of the largest TB-designated hospitals in China. Patients with newly diagnosed bacteriologically-confirmed pulmonary TB were screened for eligibility from December 1 2017 to December 31 2019. Apart from bacteriological evidence, participants should age 18–65 years and have a typical pulmonary lesion of TB by radiological examination. Those received any anti-TB treatment within 6 months preceding initiation of the study drugs; were pregnant or lactating women; had impaired liver or renal function; had a history of allergy or intolerance to any of the study drugs; were diagnosed with extra-pulmonary TB, drug-resistant TB or *nontuberculous mycobacteria* were ineligible. The detailed inclusion and exclusion criteria are presented in the online data supplement.

### Randomization and treatment allocation

Participants were randomly assigned in a 1:1 ratio either to a standard 6-month regimen group or to 4-month PRS regimen group using a random number generator. Patients and physicians were unaware of the allocated treatment until the patient had been formally entered into the trial but were not blinded afterward. Standard 6-month regimen (2HRZE/4HR) referred to the WHO treatment guidelines [13]. The PRS regimen is a four-month regimen consisting of clofazimine, prothionamide, pyrazinamide and ethambutol and has no division of intensive or consolidation phases [11]. All participants received at least one-week inpatient treatment after enrolment to closely monitor the acute adverse effects of assigned drug regimen. Directly Observed Therapy was implemented by study nurses during the in-patient phase and by community healthcare workers during the outpatient phase. Treatment adherence was evaluated at each month visits on the basis of patient’s self-report on missing doses and count of remaining tablets. The drug doses are summarized in Table E1 (see online data supplement).

### Efficacy and safety monitoring

Both groups had clinical visits before and monthly after the initiation of anti-TB treatment. Sputum smear microscopy, bacterial culture, routine blood tests, liver and renal function tests were performed at each visit, while a CT scan was obtained every two months. After treatment completion, additional follow-up was performed at 3, 6 and 12 months to evaluate the status of TB using bacterial culture and/or a CT scan. A structured case report form was used to collect demographic, clinical and laboratory information by trained research physicians. The details of microbiological work are presented in the online data supplement.

### Outcome measures

The primary efficacy outcome was the proportion of patients who achieved culture conversion while on allocated treatment, i.e. within 4 months for PRS regimen and 6 months for the standard regimen. Culture conversion was defined as having at least two consecutive negative cultures taken at least 30 days apart [14]. Secondary clinical outcomes were as follows: (1) two-month sputum culture negative conversion; (2) time to culture conversion; (3) treatment outcome according to the new definitions released by the WHO [15]; (4) early bactericidal activity (EBA); (5) radiological changes by comparing the end of treatment or last available exanimation with the baseline. Patients whose lung cavities and lesions were stable or extended compared to the baseline were defined as unchanged or deteriorated; patients whose pulmonary cavities were closed and whose lung lesions were stably absorbed by more than a half were defined as recovered; while the rest with pulmonary cavities or lung lesions absorbed by less than a half were defined as improved. The primary safety outcome was the incidence of grade 3 or 4 adverse events during anti-TB treatment, which was defined according to the Division of Acquired Immunodeficiency Syndrome (DAIDS) guidelines [16]. Permanent change of assigned regimen due to adverse drug reaction was also assessed as an important safety outcome in both regimen groups.

### Statistical analysis

Since pre-study calculation of sample size was not performed, a *post-hoc* power analysis was provided for the primary outcome. The primary and secondary analyses on efficacy were performed both in the modified intention-to-treat and per-protocol populations. The modified intention-to-treat population included all randomized patients except those who had evidence of a *nontuberculous mycobacteria*, drug-resistant *M. tuberculosis* or extrapulmonary infection, or who requested for withdrawal before starting assigned treatment. The per-protocol population was defined as a subset of the modified intention-to-treat population who completed the assigned treatment course unless the reason for inadequate treatment was death or poor treatment response. Safety analysis was performed in all randomized patients receiving at least one dose of the trial medication.

Baseline data are reported as proportions for categorical variables; as mean ± standard deviation (SD) for normally distributed data; or as median with interquartile (IQR) for nonnormally distributed continuous variables. The primary and secondary outcomes were compared between the two treatment groups using Chi-square or Fisher’s exact test, or by Wilcoxon rank-sum test, as appropriate, at a two-sided significance level of 0.05. Kaplan–Meier estimates for the distribution of time to culture conversion by treatment group were constructed, censoring data on patients who had no follow-up sputum samples, did not achieve culture conversion by the end of the treatment, permanently changed assigned regimen, were lost to follow-up or died before culture conversion occurred. Considering the nature of this pilot clinical trial, small sample size and the lack of pre-analysis plan, all results should be explained as exploratory. Analyses were performed with IBM SPSS 26.0 (IBM Corp., Armonk, NY) and R software, version 4.1.1 (R Foundation for Statistical Computing).

### Ethics consideration

This study was registered at ClinicalTrials.gov (NCT03561753) and was approved by the ethics committee of the Shanghai Pulmonary Hospital. Written informed consent was obtained from all participants before enrolment.

## Results

### Study population

**Figure 1.**
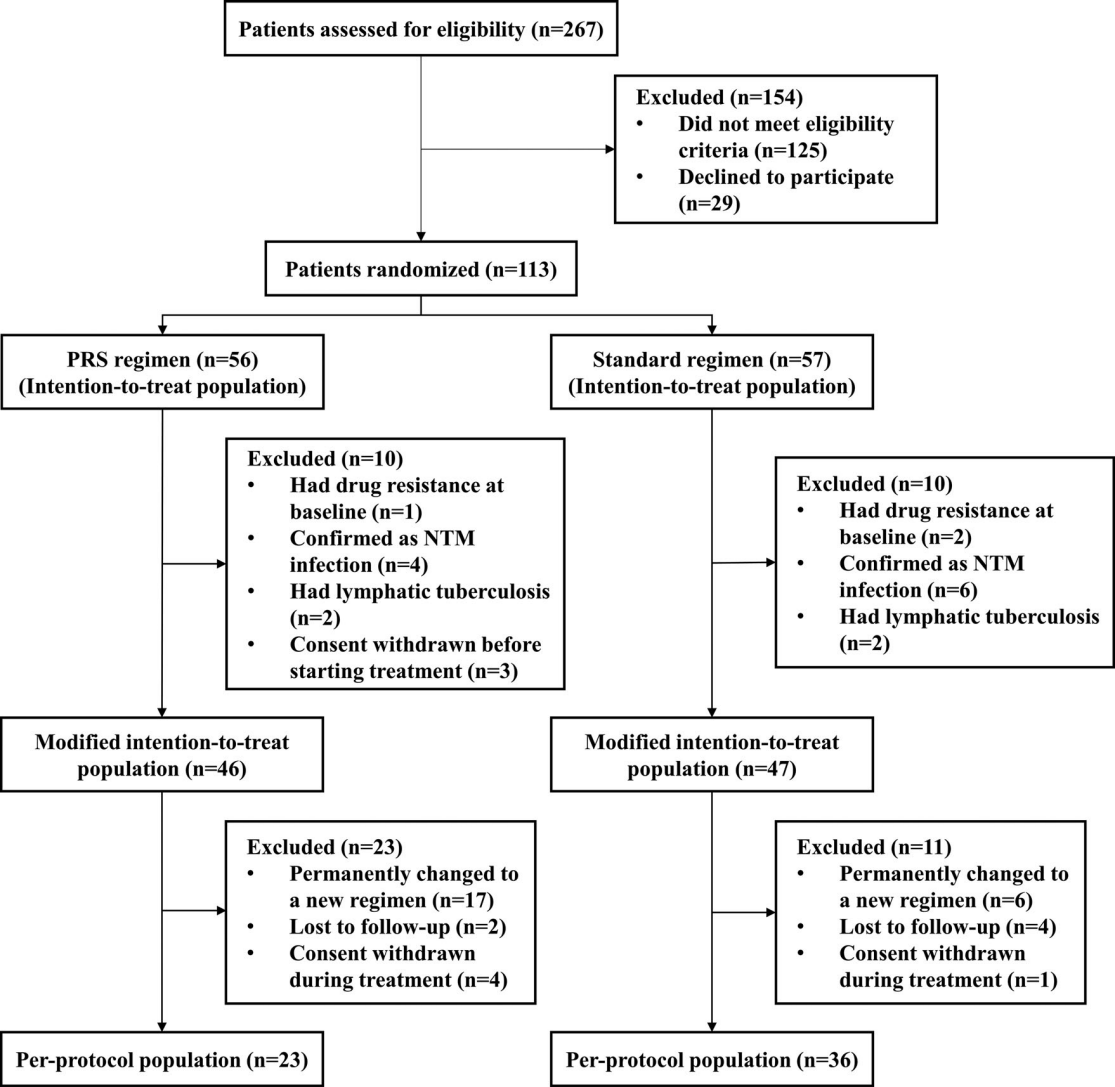
Flow chart for enrolment, randomization and follow-up of patients. One patient on the Parabolic Response Surface (PRS) regimen permanently changed assigned regimen due to shortage of clofazimine, while the rest were due to adverse drug reactions. NTM: *nontuberculous mycobacteria.*

After screening for eligibility between 1 December 2017 and 31 December 2019 in the Shanghai Pulmonary Hospital, 113 patients were randomly assigned to a treatment group. A total of 20 patients who had undergone randomization were excluded from the modified intention-to-treat population, which comprised 93 patients (46 in the short-course PRS regimen group and 47 in the standard regimen group) (Figure 1). As for per-protocol population, it comprised 59 patients, with 23 in the PRS regimen group and 36 in the standard regimen group. No self-reported missing doses or treatment interruptions were recorded for patients in the per-protocol population. The median age for the PRS regimen and standard regimen groups was 31.5 and 32.0 years, respectively, in the modified intention-to-treat population. Overall, baseline demographic and clinical characteristics of the patients were broadly comparable in two treatment groups, apart from the higher percentage of female and patients with ≥2 pulmonary cavities in standard regimen group (Table 1).

**Table 1.** Baseline characteristics of patients in the modified intention-to-treat population.

Characteristics	Standard regimen (*n* = 47)	Short-course regimen (*n* = 46)	Total (*n* = 93)
Age, yr^a^	32.0 (25.0, 49.0)	31.5 (24.8, 47.3)	32.0 (24.0, 47.0)
Sex			
Male	27 (57.4)	36 (78.3)	63 (67.7)
Female	20 (42.6)	10 (21.7)	30 (32.3)
Bodyweight, kg^a^	55.0 (50.0, 62.0)	56.0 (50.0, 63.3)	56.0 (50.0, 62.0)
No. of infected pulmonary zones^a^	4 (2, 5)	4 (3, 6)	4 (3, 5)
No. of pulmonary cavities			
0	13 (27.7)	16 (34.8)	29 (31.2)
1	11 (23.4)	18 (39.1)	29 (31.2)
≥2	23 (48.9)	12 (26.1)	35 (37.6)
Smear grade^b^			
Negative	1 (2.2)	5 (10.9)	6 (6.5)
Scanty and 1+	16 (34.0)	14 (30.4)	30 (32.2)
2+	16 (34.0)	9 (19.6)	25 (26.9)
3 + and 4+	14 (29.8)	18 (39.1)	32 (34.4)
Body-mass index^a^	19.5 (18.0, 20.7)	19.6 (18.0, 21.6)	19.5 (18.0, 21.1)
Erythrocyte sedimentation rate, mm/h^a^	59.5 (34.3, 85.8)	39.0 (24.5, 73.0)	50.0 (27.5, 83.5)
Comorbidity	5 (10.6)	2 (4.3)	7 (7.5)
Diabetes mellitus type 2	1 (2.1)	0 (0.0)	1 (1.1)
COPD	0 (0.0)	1 (2.2)	1 (1.1)
Rheumatoid arthritis	1 (2.1)	0 (0.0)	1 (1.1)
Bronchiectasis	3 (6.4)	1 (2.2)	4 (4.3)

Note: All data were presented as number (percentage) unless specified. COPD: chronic obstructive pulmonary disease.

^a^ Median (interquartile range).

^b^ Smear grade is referred to the Chinese national guidelines released in 2021 [29], which is slightly different from the World Health Organization guidelines.

### Sputum culture negative conversion

In the primary analyses of culture conversion at the end of treatment course, 20 patients were not assessable due to permanent change to a new regimen caused by adverse drug reaction (*n* = 11), consent withdrawn during treatment (*n* = 4), non-TB-specific death (*n* = 1), loss to follow-up (*n* = 2) or missing sputum samples (*n* = 2). By contrast, 17 patients achieved culture negative conversion before permanently changing assigned regimen for reasons of adverse drug reaction (n = 11) or clofazimine shortage (*n* = 1), consent withdrawn (*n* = 1) and loss to follow-up (*n* = 4), thus were considered with assessable results. In the modified intention-to-treat population, patients who received the PRS regimen and standard regimen had respective negative conversion rates of 65.2% and 87.2%, for an absolute difference of 22.0 percentage points (95% CI, 5.3–38.8; *P *= .012). However, comparable negative conversion rates were observed between patients on different regimens in per-protocol population with an absolute difference of 7.5 percentage points (95% CI, −11.7 to 26.7; *P *= .369) (Table 2).

**Table 2.** Primary efficacy analysis in the modified intention-to-treat and per-protocol populations.

Sputum culture result at the end of treatment	Modified intention-to-treat population			Per-protocol population		
	Standard regimen (*n* = 47)	PRS regimen (*n* = 46)	Total (*n* = 93)	Standard regimen (*n* = 36)	PRS regimen (*n* = 23)	Total (*n* = 59)
*Not assessable*	5 (10.6)	15 (32.6)	20 (21.5)	1 (2.8)	2 (8.7)	3 (5.1)
Due to permanent change to a new regimen caused by adverse drug reaction^a^	4 (8.5)	7 (15.3)	11 (11.7)	NA	NA	NA
Consent withdrawn during treatment^a^	0 (0.0)	4 (8.7)	4 (4.3)	NA	NA	NA
Due to loss to follow-up^a^	0 (0.0)	2 (4.3)	2 (2.2)	NA	NA	NA
Due to death^a^	1 (2.1)	0 (0.0)	1 (1.1)	1 (2.8)	0 (0.0)	1 (1.7)
Due to missing sputum samples	0 (0.0)	2 (4.3)	2 (2.2)	0 (0.0)	2 (8.7)	2 (3.4)
*Assessable*	42 (89.4)	31 (67.4)	73 (78.5)	35 (97.2)	21 (91.3)	56 (94.9)
Positive	1 (2.1)	1 (2.2)	2 (2.2)	1 (2.8)	1 (4.3)	2 (3.4)
Negative	41 (87.2)	30 (65.2)	71 (76.3)	34 (94.4)	20 (87.0)	54 (91.5)
Percentage-point difference from standard regimen in rate of negative conversion (95%CI)	NA	22.0 (5.3–38.8)	NA	NA	7.5 (−11.7 to 26.7)	NA
*P* value	NA	.012	NA	NA	0.369	NA

Note: All data were presented as number (percentage) unless specified. PRS: Parabolic Response Surface; NA: not applicable.

^a^ Occurred before the collection of first follow-up sputum samples.

**Figure 2.**
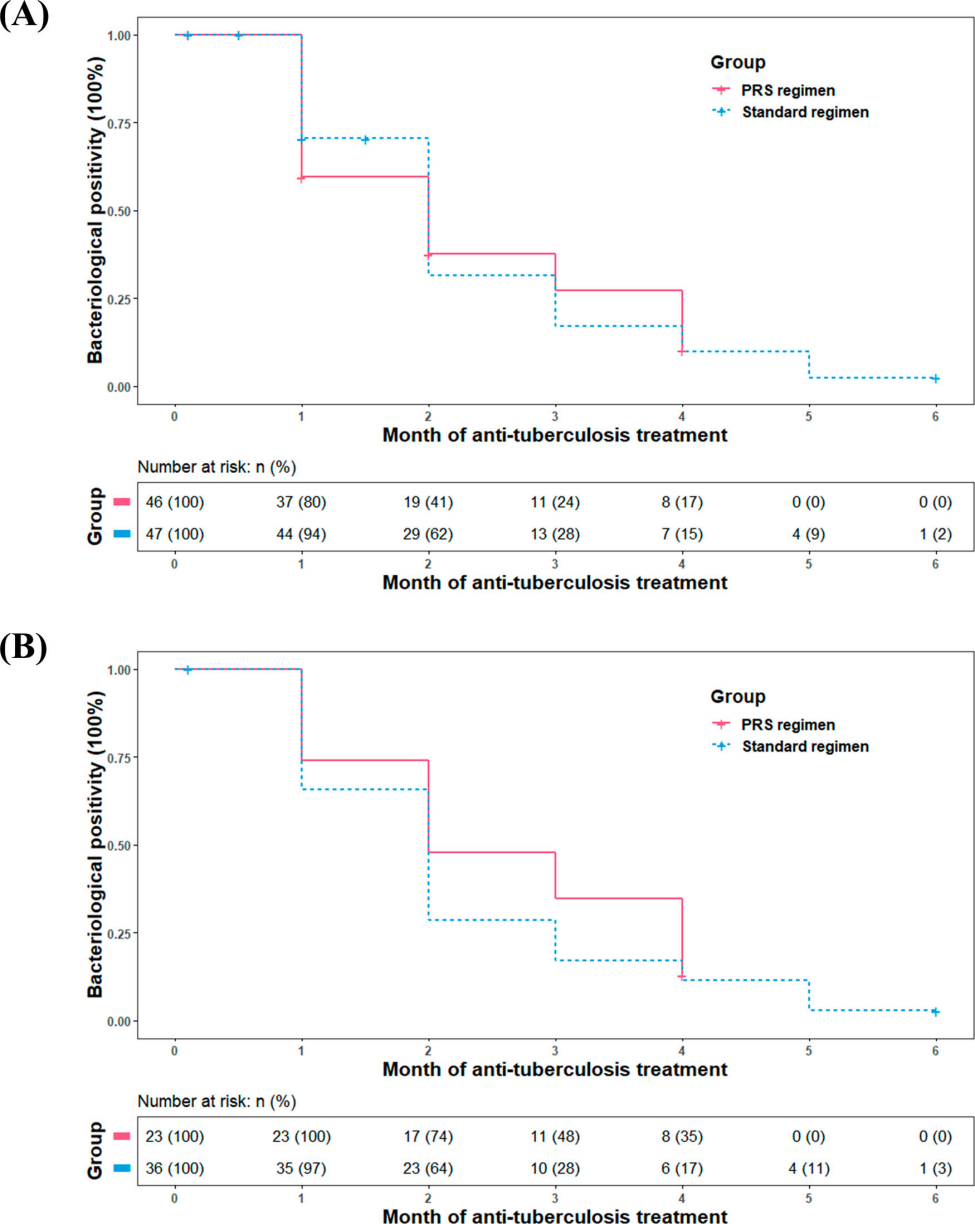
Kaplan–Meier analysis of time to culture conversion in the modified intention-to-treat population (A) (*n* = 93) and per-protocol population (B) (*n* = 59). The risk table showed the number and percentage of patients at risk of a positive sputum culture at the beginning of each month. No significant differences were observed between patients on Parabolic Response Surface (PRS) or standard regimen (*P* = .928 for modified intention-to-treat population; *P* = .298 for per-protocol population).

As shown in Table 3, there was no significant difference on two-month culture conversion rates between patients received the PRS and standard regimen neither in the modified intention-to-treat (47.8% vs. 61.7%, *P *= .179) or per-protocol populations (52.2% vs. 69.5%, *P *= .181). Meanwhile, no significant between-group differences on time to culture conversion were identified in the modified intention-to-treat (median time: 1 vs. 2 months, *P *= .928) nor per-protocol population (2 vs. 2 months, *P *= .298) (Figure 2).

**Table 3.** Secondary efficacy analysis in the modified intention-to-treat and per-protocol populations.

	Modified intention-to-treat population			Per-protocol population		
	Standard regimen (*n* = 47)	PRS regimen (*n* = 46)	Total (*n* = 93)	Standard regimen (*n* = 36)	PRS regimen (*n* = 23)	Total (*n* = 59)
**Two-month sputum culture result**						
Not assessable	11 (23.4)	19 (41.3)	30 (32.3)	4 (11.1)	6 (26.1)	10 (16.9)
Due to permanent change to a new regimen caused by adverse drug reaction^a^	4 (8.5)	7 (15.3)	11 (11.8)	NA	NA	NA
Consent withdrawn during treatment^a^	0 (0.0)	4 (8.7)	4 (4.3)	NA	NA	NA
Due to loss to follow-up^a^	0 (0.0)	2 (4.3)	2 (2.2)	NA	NA	NA
Due to death^a^	1 (2.1)	0 (0.0)	1 (1.1)	1 (2.8)	0 (0.0)	1 (1.7)
Due to missing or contaminated sputum samples at two months	6 (12.8)	6 (13.0)	12 (12.9)	3 (8.3)	6 (26.1)	9 (15.3)
Assessable	36 (76.6)	27 (58.7)	63 (67.7)	32 (88.9)	17 (73.9)	49 (83.1)
Positive	7 (14.9)	5 (10.9)	12 (12.9)	7 (19.4)	5 (21.7)	12 (20.3)
Negative	29 (61.7)	22 (47.8)	51 (54.8)	25 (69.5)	12 (52.2)	37 (62.8)
Percentage-point difference from standard regimen in rate of negative conversion (95%CI)	NA	13.9 (−6.2 to 33.9)	NA	NA	17.3 (−8.1 to 42.6)	NA
*P* value	NA	0.179	NA	NA	0.181	NA
**Radiological change^b^**						
Not assessable	8 (17.0)	20 (43.5)	28 (30.1)	2 (5.6)	1 (4.3)	3 (5.1)
Due to permanent change to a new regimen caused by adverse drug reaction^a^	5 (10.6)	13 (28.3)	18 (19.4)	NA	NA	NA
Consent withdrawn during treatment^a^	0 (0.0)	4 (8.7)	4 (4.3)	NA	NA	NA
Due to loss to follow-up^a^	0 (0.0)	2 (4.3)	2 (2.2)	NA	NA	NA
Due to death^a^	1 (2.1)	0 (0.0)	1 (1.1)	1 (2.8)	0 (0.0)	1 (1.7)
Due to missing examination	2 (4.3)	1 (2.2)	3 (3.2)	1 (2.8)	1 (4.3)	2 (3.4)
Assessable	39 (83.0)	26 (56.5)	65 (69.9)	34 (94.4)	22 (95.7)	56 (94.9)
Unchanged or deteriorated	0 (0.0)	1 (2.2)	1 (1.1)	0 (0.0)	1 (4.3)	1 (1.7)
Improved or recovered	39 (83.0)	25 (54.3)	64 (68.8)	34 (94.4)	21 (91.4)	55 (93.2)
Percentage-point difference from standard regimen in rate of improved or recovered radiological examination (95%CI)	NA	28.7 (10.7–46.6)	NA	NA	3.1 (−13.7 to 20.0)	NA
*P* value	NA	0.003	NA	NA	0.639	NA
**Treatment outcome**						
Sustained treatment success	33 (70.2)	19 (41.3)	52 (55.9)	33 (91.7)	19 (82.6)	52 (88.1)
Cure	27 (57.4)	12 (26.1)	39 (41.9)	27 (75.0)	12 (52.2)	39 (66.1)
Treatment completion	6 (12.8)	7 (15.2)	13 (14.0)	6 (16.7)	7 (30.4)	13 (22.0)
Unfavourable outcome	14 (29.8)	27 (58.7)	41 (44.1)	3 (8.3)	4 (17.4)	7 (11.9)
Treatment failed due to no treatment response	1 (2.1)	1 (2.2)	2 (2.1)	1 (2.8)	1 (4.3)	2 (3.4)
Treatment failed due to permanent change to a new regimen caused by adverse drug reaction	6 (12.9)	16 (34.8)	22 (23.6)	NA	NA	NA
Treatment failed due to permanent change to a new regimen caused by shortage of clofazimine	0 (0.0)	1 (2.2)	1 (1.1)	NA	NA	NA
Consent withdrawn during treatment	1 (2.1)	4 (8.7)	5 (5.4)	NA	NA	NA
Lost to follow-up	4 (8.5)	2 (4.3)	6 (6.5)	NA	NA	NA
Death	1 (2.1)	0 (0.0)	1 (1.1)	1 (2.8)	0 (0.0)	1 (1.7)
Recurrence	1 (2.1)	3 (6.5)	4 (4.3)	1 (2.8)	3 (13.0)	4 (6.8)
Percentage-point difference from standard regimen in rate of treatment success (95%CI)	NA	28.9 (9.6–48.2)	NA	NA	9.1 (−12.4 to 30.6)	NA
*P* value	NA	.005	NA	NA	0.415	NA

All data were presented as number (percentage) unless specified. PRS: Parabolic Response Surface; NA: not applicable.

^a^ Occurred before the collection of first follow-up sputum samples or first radiological examination.

^b^ By comparing the end of treatment or last available exanimation with the baseline.

### Treatment outcome

Analysis of treatment outcomes in the modified intention-to-treat population showed that a sustained treatment success occurred in 41.3% (19/46) and 70.2% (33/47) of the patients who received the PRS regimen and standard regimen, respectively, for an absolute difference of 28.9 percentage points (95% CI, 9.6–48.2; *P *= 0.005). The unfavourable outcomes in the PRS group were mainly attributed to permanent regimen change as a result of adverse drug reaction (34.8% vs. 12.9%), primarily drug-induced hepatitis. In per-protocol population, comparable efficacy was observed for the PRS and standard regimen groups for an absolute difference of 9.1 percentage points (95% CI, −12.4 to 30.6; *P *= .415). The median time for post-treatment follow-up was 12 months in patients with treatment success, of whom 80.4% (45/56) completed one-year follow-up after treatment. In total, recurrence of TB was observed for four patients, with three in the PRS regimen group and one in the standard regimen group.

### Other secondary clinical outcomes

**Figure 3.**
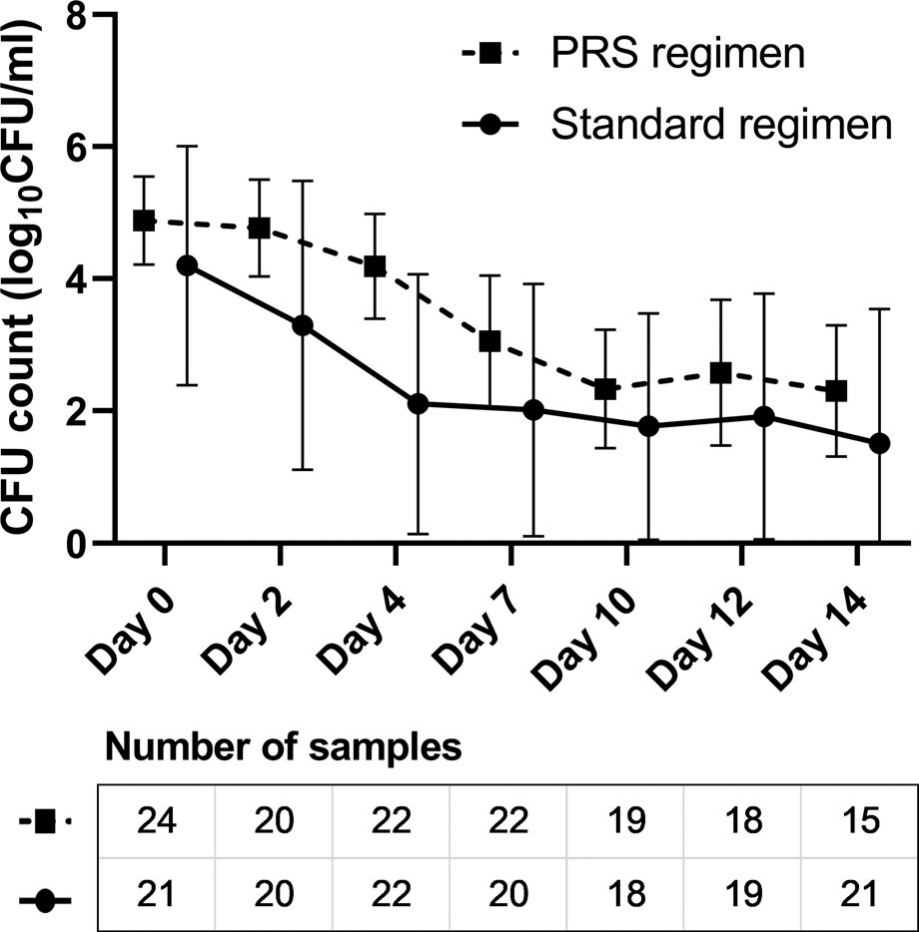
Early bactericidal activity (EBA) of the standard and Parabolic Response Surface (PRS) regimens in the modified intention-to-treat population with assessable results. The number of sputum samples tested at each time point are presented in the figures, after excluding the missing (mainly due to the difficulty of expectorating), contaminated or low-quality sputum samples. No significant between-group differences were identified on EBA_0-2 days_ (median: 0.27 vs. 0.46 log_10_ CFU ml^−1^ d^−1^, *P *= .177), EBA_2-14 days_ (0.19 vs. 0.10 log_10_ CFU ml^−1^ d^−1^, *P *= .182), nor EBA_0-14 days_ (0.19 vs. 0.22 log_10_ CFU ml^−1^ d^−1^, *P *= .739).

EBA analysis was performed among patients with assessable test results in the modified intention-to-treat population after excluding those whose samples were contaminated, who had difficulty in expectorating, or had low-quality sputum samples. As shown in Figure 3, the EBA_0-2 days_ (median value: 0.27 vs. 0.46 log_10_ CFU ml^−1^ d^−1^, *P *= .177), EBA_2-14 days_ (0.19 vs. 0.10 log_10_ CFU ml^−1^ d^−1^, *P *= .182) and EBA_0-14 days_ (0.19 vs. 0.22 log_10_ CFU ml^−1^ d^−1^, *P *= .739) were comparable between the PRS and standard regimen groups. The results of radiological change showed that the PRS regimen group had lower proportion of improved or recovered radiological presentation in the modified intention-to-treat population (54.3% vs. 83.0%, *P *= .003), but mainly due to a high proportion of not assessable results (43.5% vs. 17.0%). Per-protocol analysis, which minimizes the influence of not assessable results, showed that two treatment groups had comparable rates of radiological improvement and recovery (91.4% vs. 94.4%, *P *= .639) (Table 3).

## Adverse event

Three patients on the PRS regimen were excluded from safety analysis because consent was withdrawn before starting treatment (Figure 1). In all randomized patients receiving at least one dose of trial medication, the incidence rates of grade 3 or higher adverse events were insignificantly different for the PRS and standard regimen groups [39.6% (21/53) vs. 38.6% (22/57), *P *= .912]. Specifically, the main adverse events in the PRS regimen group were drug-induced hepatitis (61.9%, 13/21), and 92.3% (12/13) of them occurred in the first two months of treatment. For patients on the standard regimen, the main adverse events were hyperuricemia (81.8%, 18/22). To be noted, two patients in the PRS regimen group had combined elevations of transaminases (> 3 upper limit of normal) and total bilirubin (> 2 upper limit of normal), fulfilling the Hy’s criteria for serious liver injury [17]. However, permanent change of assigned regimen was more commonly seen in patients on the PRS regimen than standard regimen, for an absolute difference of 19.8 percentage points (95% CI, 4.6–35.0; *P *= .012). The main cause for the change of assigned regimen was drug-induced hepatitis (18.2%, 20/110), which occurred more frequently in the PRS regimen group (30.2% vs. 7.0%) (Table 4).

**Table 4.** Safety analysis in all randomized patients receiving at least one dose of the trial medication.

	Standard regimen (*n* = 57)	PRS regimen (*n* = 53)	Total (*n* = 110)
**Primary safety outcome**			
Grade 3 or higher adverse event	22 (38.6)	21 (39.6)	43 (39.1)
Percentage-point difference from standard regimen in rate of Grade 3 or higher adverse event (95%CI)	NA	−1.0 (−19.3 to 17.2)	NA
*P* value	NA	0.912	NA
**Other safety outcomes**			
ALT or AST level ≥5 × ULN	6 (10.5)	13 (23.2)	19 (16.8)
ALT or AST level ≥10 × ULN	3 (5.3)	6 (11.3)	9 (8.2)
TBIL level ≥2.6 × ULN	1 (1.8)	2 (3.8)	3 (2.7)
TBIL level ≥5 × ULN	0 (0.0)	2 (3.8)	2 (1.8)
UA level ≥5 × ULN	18 (31.6)	10 (18.9)	28 (25.5)
UA level ≥10 × ULN	2 (3.5)	4 (7.5)	6 (5.5)
**Permanent change of assigned regimen due to adverse drug reaction**	7 (12.3)	17 (32.1)	24 (21.8)
Hepatitis	4 (7.0)	16 (30.2)	20 (18.2)
Fever	2 (3.5)	1 (1.9)	3 (2.7)
Rash	1 (1.8)	0 (0.0)	1 (0.9)
Percentage-point difference from standard regimen in rate of permanent change of assigned regimen (95%CI)	NA	−19.8 (−35.0 to −4.6)	NA
*P* value	NA	.012	NA

Notes: All data were presented as number (percentage) unless specified. PRS: Parabolic Response Surface; ALT: alanine aminotransferase; AST: aspartate aminotransferase; TBIL: total bilirubin; UA: uric acid; ULN: upper limit of normal; NA: not applicable.

## Discussion

In this open-label, pilot randomized clinical trial, the short-course (4-month) PRS regimen consisting of clofazimine, prothionamide, pyrazinamide and ethambutol appeared to have inferior efficacy in the modified intention-to-treat population and higher incidence of drug-induced hepatitis. Its inferior efficacy is mainly attributed to the higher rates of permanent regimen change due to adverse drug reactions and consent withdrawn during treatment. As per-protocol analysis showed, both groups achieved high rates of culture conversion, radiological improvement and sustained treatment success (all above 80%).

Considering the complex drug mechanism of action and drug–drug interactions, how to properly select and combine available drugs for multidrug chemotherapy is always a question in clinical practice. Design of an optimal drug-dose combination among currently available drugs is challenging because of the exponentially large number of possible drug-dose combinations [12]. To overcome this barrier, the research team at the University of California at Los Angeles has proposed an output-driven PRS (more recently referred to as the Phenotypic Response Surface) approach to effectively identify optimal combinations among billions of drug-dose possibilities [12,18]. The clinical findings from this study were consistent with our previous work in *M. tuberculosis*-infected mice [11]. A major advantage of the PRS regimen studied here is that it is comprised of exclusively generic drugs. Regions of the world with a shortage of rifapentine, a low prevalence of resistance to pyrazinamide or a capacity to perform phenotypic drug susceptibility testing for pyrazinamide might benefit from this PRS regimen, while higher risk of adverse drug reaction should be kept in mind [6].

Although not entirely unanticipated, the high incidence of drug-induced hepatitis, the main cause for permanent change of assigned regimen in this study, in the PRS regimen group was still surprising since neither clofazimine nor prothionamide are the usual TB drugs causing hepatotoxicity [5,19,20]. It should be no more difficult and probably less difficult to manage patients on the PRS regimen than those on the standard regimen, which comprises three hepatotoxic drugs - isoniazid, rifampicin and pyrazinamide [13]. Concerns may arise with respect to the possibility of unanticipated complex drug–drug interactions among clofazimine, prothionamide, pyrazinamide and ethambutol, but these four drugs have been frequently used as a combination for multidrug-resistant or extensively drug-resistant TB treatment and no increased risk of hepatotoxicity has been reported [22]. As noted, preclinical studies in mice indicated that the efficacy of the PRS regimen was insensitive to a lowering dose of prothionamide over this dose range, but sensitive to a lowering dose of pyrazinamide [11]. For this reason, the protocol anticipated the potential risk of hepatotoxicity and allowed for (a) the sequential lowering of the prothionamide dose by 1/3, 1/2, or 2/3 at the discretion of the attending physician; (b) or to change the assigned PRS regimen. Nevertheless, the attending physician elected to exercise the latter option. In any future clinical trials of the PRS regimen, adverse drug reactions should be thoroughly considered during study preparation and be responded accordingly.

There are several limitations of our study. Firstly, most comparisons in this study were underpowered and inconclusive due to the limited sample size. The *post-hoc* power analysis showed that this study had a 72.4% power, at a two-sided 0.05 significance level, of detecting a significant difference in the rate of sputum culture negative conversion between the PRS (65.2%) and standard (87.2%) regimen groups. Meanwhile, though it was a randomized controlled trial, chance imbalances between groups did occur, such as more advanced pulmonary cavitation in patients on standard regimen. As it is a pilot study with a small sample size, multivariable analyses were not performed for adjustment. Hence, all results should be explained as exploratory. Secondly, as an open-label trial, there might be a higher risk of attrition bias and assessor bias [23]. For instance, more patients requested to withdraw before or during treatment in the PRS regimen group. Regarding assessor bias, objective criteria should be rigorously implemented in future trials to control for it [24]. Thirdly, drug susceptibility testing was not performed for clofazimine, prothionamide and pyrazinamide in this study due to the lack of a sufficiently reliable method [25–27] and logistical hurdles. Considering pyrazinamide is one of the cornerstone drugs in the PRS regimen, drug susceptibility testing is recommended before initiating treatment when quality assured laboratory is available. Finally, since whole genome sequencing was not performed, relapse could not be distinguished from reinfection in this study.

Preclinical studies conducted subsequent to the start of this pilot human study identified much more potent PRS regimens than the one studied here. These ultra short-course regimens reduced the time needed to achieve relapse-free cure in mice by up to 85% compared with the standard regimen [11,27–29]. In contrast to the PRS regimen studied here, the ultra short-course PRS regimens include newly approved drugs such as bedaquiline and additionally either the new drug delamanid or the experimental drug SQ109. Future clinical studies are planned to evaluate these new ultra short-course PRS regimens.

In conclusion, the 4-month PRS regimen is a completely new drug combination for drug-susceptible TB treatment, breaking the traditional concept of first- and second-line drugs. Though the PRS regimen group was observed with inferior efficacy in the modified intention-to-treat population, both treatment groups appeared to have desired efficacy in per-protocol population. The unanticipated high incidence of drug-induced hepatitis in PRS regimen group underlines the importance of safety assessment for proof-of-concept studies. While future clinical studies are more likely to focus on newer more potent PRS regimens that include non-generic drugs, this study provides the first proof-of-concept in humans that the artificial intelligence-enabled PRS approach can identify drug regimens with the capacity to markedly shorten the duration of treatment required to cure patients with TB.

## Supplementary Material

Supplemental Material
